# Metacognition: ideas and insights from neuro- and educational sciences

**DOI:** 10.1038/s41539-021-00089-5

**Published:** 2021-06-08

**Authors:** Damien S. Fleur, Bert Bredeweg, Wouter van den Bos

**Affiliations:** 1 Informatics Institute, University of Amsterdam, Amsterdam, the Netherlands; 2 Departement of Psychology, University of Amsterdam, Amsterdam, the Netherlands; 3 Faculty of Education, Amsterdam University of Applied Sciences, Amsterdam, the Netherlands; 4 Center for Adaptive Rationality, Max Planck Institute for Human Development, Berlin, Germany

**Keywords:** Education, Human behaviour, Education, Interdisciplinary studies

## Abstract

Metacognition comprises both the ability to be aware of one’s cognitive processes (metacognitive knowledge) and to regulate them (metacognitive control). Research in educational sciences has amassed a large body of evidence on the importance of metacognition in learning and academic achievement. More recently, metacognition has been studied from experimental and cognitive neuroscience perspectives. This research has started to identify brain regions that encode metacognitive processes. However, the educational and neuroscience disciplines have largely developed separately with little exchange and communication. In this article, we review the literature on metacognition in educational and cognitive neuroscience and identify entry points for synthesis. We argue that to improve our understanding of metacognition, future research needs to (i) investigate the degree to which different protocols relate to the similar or different metacognitive constructs and processes, (ii) implement experiments to identify neural substrates necessary for metacognition based on protocols used in educational sciences, (iii) study the effects of training metacognitive knowledge in the brain, and (iv) perform developmental research in the metacognitive brain and compare it with the existing developmental literature from educational sciences regarding the domain-generality of metacognition.

## Introduction

Metacognition is defined as “thinking about thinking” or the ability to monitor and control one’s cognitive processes^1^ and plays an important role in learning and education^2–4^. For instance, high performers tend to present better metacognitive abilities (especially control) than low performers in diverse educational activities^5–9^. Recently, there has been a lot of progress in studying the neural mechanisms of metacognition^10,11^, yet it is unclear at this point how these results may inform educational sciences or interventions. Given the potential benefits of metacognition, it is important to get a better understanding of how metacognition works and of how training can be useful.

The interest in bridging cognitive neuroscience and educational practices has increased in the past two decades, spanning a large number of studies grouped under the umbrella term of educational neuroscience^12–14^. With it, researchers have brought forward issues that are viewed as critical for the discipline to improve education. Recurring issues that may impede the relevance of neural insights for educational practices concern external validity^15,16^, theoretical discrepancies^17^ and differences in terms of the domains of (meta)cognition operationalised (specific or general)^15^. This is important because, in recent years, brain research is starting to orient itself towards training metacognitive abilities that would translate into real-life benefits. However, direct links between metacognition in the brain and metacognition in domains such as education have still to be made. As for educational sciences, a large body of literature on metacognitive training is available, yet we still need clear insights about what works and why. While studies suggest that training metacognitive abilities results in higher academic achievement^18^, other interventions show mixed results^19,20^. Moreover, little is known about the long-term effects of, or transfer effects, of these interventions. A better understanding of the cognitive processes involved in metacognition and how they are expressed in the brain may provide insights in these regards.

Within cognitive neuroscience, there has been a long tradition of studying executive functions (EF), which are closely related to metacognitive processes^21^. Similar to metacognition, EF shows a positive relationship with learning at school. For instance, performance in laboratory tasks involving error monitoring, inhibition and working memory (i.e. processes that monitor and regulate cognition) are associated with academic achievement in pre-school children^22^. More recently, researchers have studied metacognition in terms of introspective judgements about performance in a task^10^. Although the neural correlates of such behaviour are being revealed^10,11^, little is known about how behaviour during such tasks relates to academic achievement.

Educational and cognitive neuroscientists study metacognition in different contexts using different methods. Indeed, while the latter investigate metacognition via behavioural task, the former mainly rely on introspective questionnaires. The extent to which these different operationalisations of metacognition match and reflect the same processes is unclear. As a result, the external validity of methodologies used in cognitive neuroscience is also unclear^16^. We argue that neurocognitive research on metacognition has a lot of potential to provide insights in mechanisms relevant in educational contexts, and that theoretical and methodological exchange between the two disciplines can benefit neuroscientific research in terms of ecological validity.

**Fig. 1:**
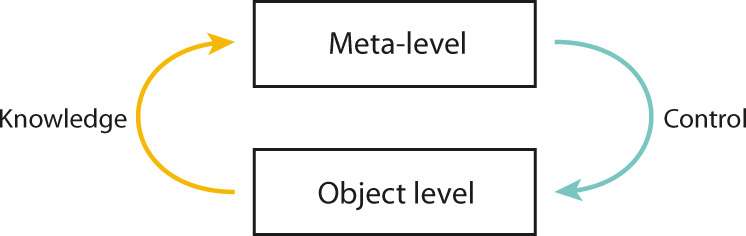
Model of metacognitive processes. Meta-knowledge is characterised as the upward flow from object-level to meta-level. Meta-control is characterised as the downward flow from meta-level to object-level. Metacognition is therefore conceptualised as the bottom-up monitoring and top-down control of object-level processes. Adapted from Nelson and Narens’ cognitive psychology model of metacognition^23^.

For these reasons, we investigate the literature through the lenses of external validity, theoretical discrepancies, domain generality and metacognitive training. Research on metacognition in cognitive neuroscience and educational sciences are reviewed separately. First, we investigate how metacognition is operationalised with respect to the common framework introduced by Nelson and Narens^23^ (see Fig. 1). We then discuss the existing body of evidence regarding metacognitive training. Finally, we compare findings in both fields, highlight gaps and shortcomings, and propose avenues for research relying on crossovers of the two disciplines.

In cognitive neuroscience, metacognition is divided into two main components^5,24^, which originate from the seminal works of Flavell on metamemory^25,26^. First, metacognitive knowledge (henceforth, meta-knowledge) is defined as the knowledge individuals have of their own cognitive processes and their ability to monitor and reflect on them. Second, metacognitive control (henceforth, meta-control) consists of someone’s self-regulatory mechanisms, such as planning and adapting behaviour based on outcomes^5,27^. Following Nelson and Narens’ definition^23^, meta-knowledge is characterised as the flow and processing of information from the object level to the meta-level, and meta-control as the flow from the meta-level to the object level^28–30^ (Fig. 1). The object-level encompasses cognitive functions such as recognition and discrimination of objects, decision-making, semantic encoding, and spatial representation. On the meta-level, information originating from the object level is processed and top-down regulation on object-level functions is imposed^28–30^.

Educational researchers have mainly investigated metacognition through the lens of *Self-Regulated Learning* theory (SRL)^3,4^, which shares common conceptual roots with the theoretical framework used in cognitive neuroscience but varies from it in several ways^31^. First, SRL is constrained to learning activities, usually within educational settings. Second, metacognition is merely one of three components, with “motivation to learn” and “behavioural processes”, that enable individuals to learn in a self-directed manner^3^. In SRL, metacognition is defined as setting goals, planning, organising, self-monitoring and self-evaluating “at various points during the acquisition”^3^. The distinction between meta-knowledge and meta-control is not formally laid down although reference is often made to a “self-oriented feedback loop” describing the relationship between reflecting and regulating processes that resembles Nelson and Narens’ model (Fig. 1)^3,23^. In order to facilitate the comparison of operational definitions, we will refer to meta-knowledge in educational sciences when protocols operationalise self-awareness and knowledge of strategies, and to meta-control when they operationalise the selection and use of learning strategies and planning. For an in-depth discussion on metacognition and SRL, we refer to Dinsmore et al. ^31^.

## Metacognition in cognitive neuroscience

### Operational definitions

In cognitive neuroscience, research in metacognition is split into two tracks^32^. One track mainly studies meta-knowledge by investigating the neural basis of introspective judgements about one’s own cognition (i.e., metacognitive judgements), and meta-control with experiments involving cognitive offloading. In these experiments, subjects can perform actions such as set reminders, making notes and delegating tasks^33,34^, or report their desire for them^35^. Some research has investigated how metacognitive judgements can influence subsequent cognitive behaviour (i.e., a downward stream from the meta-level to the object level), but only one study so far has explored how this relationship is mapped in the brain^35^. In the other track, researchers investigate EF, also referred to as cognitive control^30,36^, which is closely related to metacognition. Note however that EF are often not framed in metacognitive terms in the literature^37^ (but see ref. ^30^). For the sake of concision, we limit our review to operational definitions that have been used in neuroscientific studies.

### Metacognitive judgements

Cognitive neuroscientists have been using paradigms in which subjects make judgements on how confident they are with regards to their learning of some given material^10^. These judgements are commonly referred to as *metacognitive judgements*, which can be viewed as a form of meta-knowledge (for reviews see Schwartz^38^ and Nelson^39^). Historically, researchers mostly resorted to paradigms known as Feelings of Knowing (FOK)^40^ and Judgements of Learning (JOL)^41^. FOK reflect the belief of a subject to knowing the answer to a question or a problem and being able to recognise it from a list of alternatives, despite being unable to explicitly recall it^40^. Here, metacognitive judgement is thus made after retrieval attempt. In contrast, JOL are prospective judgements during learning of one’s ability to successfully recall an item on subsequent testing^41^.

**Fig. 2:**
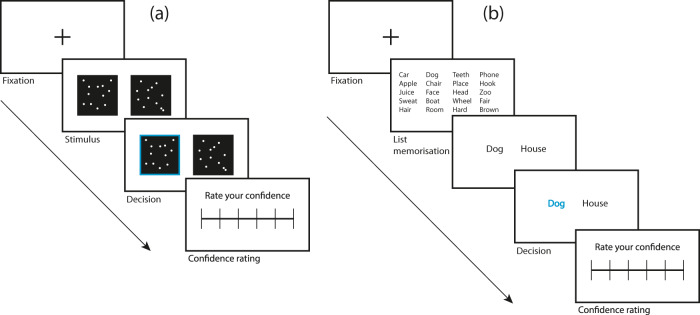
Examples of 2-AFC tasks with the addition of post-decisional confidence judgement used to assess meta-knowledge. **a** Visual perception task: subjects choose the box containing the most (randomly generated) dots. Subjects then rate their confidence in their decision. **b** Memory task: subjects learn a list of words. In the next screen, they have to identify which of two words shown was present on the list. The subjects then rate their confidence in their decision.

**Fig. 3:**
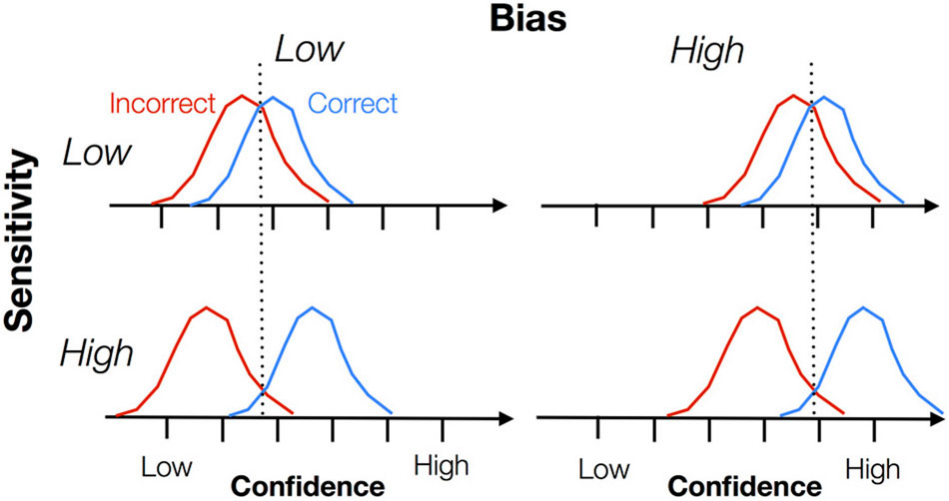
Four configurations of metacognitive sensitivity and bias levels. The red and blue curves represent the distribution of confidence ratings for incorrect and correct trials, respectively. A larger distance between the two curves denotes higher sensitivity. Displacement to the left and right denote biases towards low confidence (low metacognitive bias) and high confidence (high metacognitive bias), respectively (retrieved from Fig. 1 in Fleming and Lau^46^). We repeat the disclaimer of the original authors that this figure is not a statistically accurate description of correct and incorrect responses, which are typically not normally distributed^46,47^.

More recently, cognitive neuroscientists have used paradigms in which subjects make retrospective metacognitive judgements on their performance in a two-alternative Forced Choice task (2-AFC)^42^. In 2-AFCs, subjects are asked to choose which of two presented options has the highest criterion value. Different domains can be involved, such as perception (e.g., visual or auditory) and memory. For example, subjects may be instructed to visually discriminate which one of two boxes contains more dots^43^, identify higher contrast Gabor patches^44^, or recognise novel words from words that were previously learned^45^ (Fig. 2). The subjects engage in metacognitive judgements by rating how confident they are relative to their decision in the task. Based on their responses, one can evaluate a subject’s *metacognitive sensitivity* (the ability to discriminate one’s own correct and incorrect judgements), *metacognitive bias* (the overall level of confidence during a task), and *metacognitive efficiency* (the level of metacognitive sensitivity when controlling for task performance^46^; Fig. 3). Note that sensitivity and bias are independent aspects of metacognition, meaning that two subjects may display the same levels of metacognitive sensitivity, but one may be biased towards high confidence while the other is biased towards low confidence. Because metacognitive sensitivity is affected by the difficulty of the task (one subject tends to display greater metacognitive sensitivity in easy tasks than difficult ones and different subjects may find a task more or less easy), metacognitive efficiency is an important measure as it allows researchers to compare metacognitive abilities between subjects and between domains. The most commonly used methods to assess metacognitive sensitivity during retrospective judgements are the receiver operating curve (ROC) and meta-*d*′.^46^ Both derive from signal detection theory (SDT)^47^ which allows Type 1 sensitivity, or *d’*′ (how a subject can discriminate between stimulus alternatives, i.e. object-level processes) to be differentiated from metacognitive sensitivity (a judgement on the correctness of this decision)^48^. Importantly, only comparing meta-*d*′ to *d*′ seems to give reliable assessments metacognitive efficiency^49^. A ratio of 1 between meta-*d’*′ and *d’*′, indicates that a subject was perfectly able to discriminate between their correct and incorrect judgements. A ratio of 0.8 suggests that 80% of the task-related sensory evidence was available for the metacognitive judgements. Table 1 provides an overview of the different types of tasks and protocols with regards to the type of metacognitive process they operationalise. These operationalisations of meta-knowledge are used in combination with brain imaging methods (functional and structural magnetic resonance imaging; fMRI; MRI) to identify brain regions associated with metacognitive activity and metacognitive abilities^10,50^. Alternatively, transcranial magnetic stimulation (TMS) can be used to temporarily deactivate chosen brain regions and test whether this affects metacognitive abilities in given tasks^51,52^.

**Table 1:** Overview of popular operationalisations of metacognition.

	Online	Offline	Protocol	Discipline	
				CN	ES
Meta-knowledge		Metacognitive judgements	JOL	X	X
			FOK	X	X
			2-AFC + confidence	X	
		Self-evaluation	MAI		X
			Interviews		X
		Awareness of learning	Learning journals		X
		Self-monitoring	Thinking-aloud		X
Meta-control		Strategy selection	MAI		X
			MSLQ		X
			LASSI		X
			Learning journals		X
		Use of strategies	Interviews		X
			Thinking-aloud		X
	Conflict monitoring		Motion discrimination + additional evidence	X	
			Flanker task	X	X
			Stroop task	X	X
	Effort regulation		2-AFC + Cognitive offloading	X	
			Demand-selection task	X	
			BRIEF		X
	Inhibition		BRIEF		X
			Flanker task	X	X
	Shifting		Stroop task	X	X
	Emotional control		BRIEF		X
		Planning	MAI		X
			MSLQ		X
			LASSI		X
			BRIEF		X
		Cognitive offloading	Go/no-go + setting reminders	X	
			Go/no-go + desire for reminder	X	

*CN* cognitive neuroscience, *ES* educational sciences.

A recent meta-analysis analysed 47 neuroimaging studies on metacognition and identified a domain-general network associated with high vs. low confidence ratings in both decision-making tasks (perception 2-AFC) and memory tasks (JOL, FOK)^11^. This network includes the medial and lateral prefrontal cortex (mPFC and lPFC, respectively), precuneus and insula. In contrast, the right anterior dorsolateral PFC (dlPFC) was specifically involved in decision-making tasks, and the bilateral parahippocampal cortex was specific to memory tasks. In addition, prospective judgements were associated with the posterior mPFC, left dlPFC and right insula, whereas retrospective judgements were associated with bilateral parahippocampal cortex and left inferior frontal gyrus. Finally, emerging evidence suggests a role of the right rostrolateral PFC (rlPFC)^53,54^, anterior PFC (aPFC)^44,45,55,56^, dorsal anterior cingulate cortex (dACC)^54,55^ and precuneus^45,55^ in metacognitive sensitivity (meta-*d*′, ROC). In addition, several studies suggest that the aPFC relates to metacognition specifically in perception-related 2-AFC tasks, whereas the precuneus is engaged specifically in memory-related 2-AFC tasks^45,55,56^. This may suggest that metacognitive processes engage some regions in a domain-specific manner, while other regions are domain-general. For educational scientists, this could mean that some domains of metacognition may be more relevant for learning and, granted sufficient plasticity of the associated brain regions, that targeting them during interventions may show more substantial benefits. Note that rating one’s confidence and metacognitive sensitivity likely involve additional, peripheral cognitive processes instead of purely metacognitive ones. These regions are therefore associated with metacognition but not uniquely per se. Notably, a recent meta-analysis^50^ suggests that domain-specific and domain-general signals may rather share common circuitry, but that their neural signature varies depending on the type of task or activity, showing that domain-generality in metacognition is complex and still needs to be better understood.

In terms of the role of metacognitive judgements on future behaviour, one study found that brain patterns associated with the desire for cognitive offloading (i.e., meta-control) partially overlap with those associated with meta-knowledge (metacognitive judgements of confidence), suggesting that meta-control is driven by either non-metacognitive, in addition to metacognitive, processes or by a combination of different domain-specific meta-knowledge processes^35^.

### Executive function

**Fig. 4:**
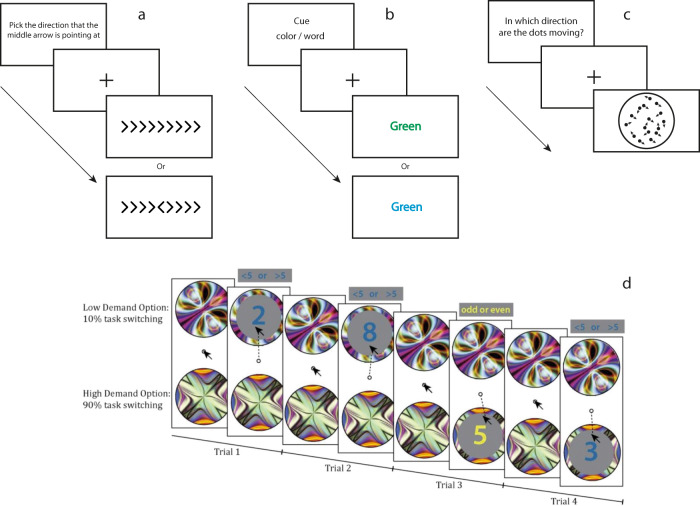
Examples of tasks used to assess EF. **a** Flanker task: subjects indicate the direction to which the arrow in the middle points. **b** Stroop task: subjects are presented with the name of colour printed in a colour that either matches or mismatches the name. Subjects are asked to give the name of the written colour or the printed colour. **c** Motion Discrimination task: subjects have to determine in which direction the dots are going with variating levels of noise. **d** Example of a Demand Selection task: in both options subjects have to switch between two tasks. Task one, subjects determine whether the number shown is higher or lower than 5. Task two, subjects determine whether the number is odd or even. The two options (low and high demand) differ in their degree of task switching, meaning the effort required. Subjects are allowed to switch between the two options. Note, the type of task is solely indicated by the colour of the number and that the subjects are not explicitly told about the difference in effort between the two options (retrieved from Fig. 1c in Froböse et al. ^58^).

In EF, processes such as error detection/monitoring and effort monitoring can be related to meta-knowledge while error correction, inhibitory control, and resource allocation can be related to meta-control^36^. To activate these processes, participants are asked to perform tasks in laboratory settings such as Flanker tasks, Stroop tasks, Demand Selection tasks and Motion Discrimination tasks (Fig. 4). Neural correlates of EF are investigated by having subjects perform such tasks while their brain activity is recorded with fMRI or electroencephalography (EEG). Additionally, patients with brain lesions can be tested against healthy participants to evaluate the functional role of the impaired regions^57^.

In a review article on the neural basis of EF (in which they are defined as meta-control), Shimamura argues that a network of regions composed of the aPFC, ACC, ventrolateral PFC (vlPFC) and dlPFC is involved in the regulations of cognition^30^. These regions are not only interconnected but are also intricately connected to cortical and subcortical regions outside of the PFC. The vlPFC was shown to play an important role in “selecting and maintaining information in working memory”, whereas the dlPFC is involved in “manipulating and updating information in working memory”^30^. The ACC has been proposed to monitor cognitive conflict (e.g. in a Stroop task or a Flanker task), and the dlPFC to regulate it^58,59^. In particular, activity in the ACC in conflict monitoring (meta-knowledge) seems to contribute to control of cognition (meta-control) in the dlPFC^60,61^ and to “bias behavioural decision-making toward cognitively efficient tasks and strategies” (p. 356)^62^ . In a recent fMRI study, subjects performed a motion discrimination task (Fig. 4c)^63^. After deciding on the direction of the motion, they were presented additional motion (i.e. post-decisional evidence) and then were asked to rate their confidence in their initial choice. The post-decisional evidence was encoded in the activity of the posterior medial frontal cortex (pMFC; meta-knowledge), while lateral aPFC (meta-control) modulated the impact of this evidence on subsequent confidence rating^63^. Finally, results from a meta-analysis study on cognitive control identified functional connectivity between the pMFC, associated with monitoring and informing other regions about the need for regulation, and the lPFC that would effectively regulate cognition^64^.

### Online vs. offline metacognition

**Fig. 5:**
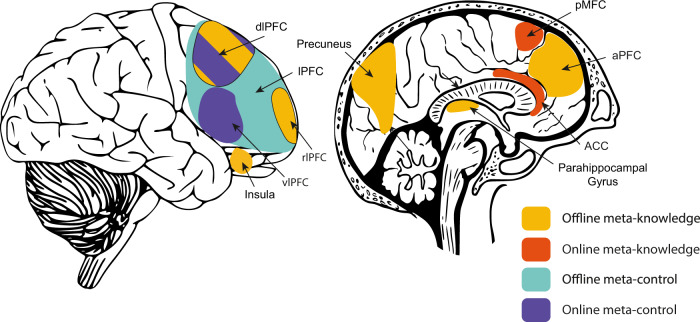
Brain regions associated with metacognition in the cognitive neuroscience literature. The regions are divided into *online* meta-knowledge and meta-control, and *offline* meta-knowledge and meta-control following the distinctions introduced earlier. Some regions have been reported to be related to both offline and online processes and are therefore given a striped pattern.

While the processes engaged during tasks such as those used in EF research can be considered as metacognitive in the sense that they are higher-order functions that monitor and control lower cognitive processes, scientists have argued that they are not functionally equivalent to metacognitive judgements^10,11,65,66^. Indeed, engaging in metacognitive judgements requires subjects to reflect on past or future activities. As such, metacognitive judgements can be considered as *offline* metacognitive processes. In contrast, high-order processes involved in decision-making tasks such as used in EF research are arguably largely made on the fly, or *online*, at a rapid pace and subjects do not *need* to reflect on their actions to perform them. Hence, we propose to explicitly distinguish *online* and *offline* processes. Other researchers have shared a similar view and some have proposed models for metacognition that make similar distinctions^65–68^. The functional difference between online and offline metacognition is supported by some evidence. For instance, event-related brain potential (ERP) studies suggest that error negativities are associated with error detection in general, whereas an increased error positivity specifically encodes error that subjects could report upon^69,70^. Furthermore, brain-imaging studies suggest that the MFC and ACC are involved in online meta-knowledge, while the aPFC and lPFC seem to be activated when subjects engage in more offline meta-knowledge and meta-control, respectively^63,71,72^. An overview of the different tasks can be found in Table 1 and a list of different studies on metacognition can be found in Supplementary Table 1 (organised in terms of the type of processes investigated, the protocols and brain measures used, along with the brain regions identified). Figure 5 illustrates the different brain regions associated with meta-knowledge and meta-control, distinguishing between what we consider to be online and offline processes. This distinction is often not made explicitly but it will be specifically helpful when building bridges between cognitive neuroscience and educational sciences.

### Training metacognition

There are extensive accounts in the literature of efforts to improve EF components such as inhibitory control, attention shifting and working memory^22^. While working memory does not directly reflect metacognitive abilities, its training is often hypothesised to improve general cognitive abilities and academic achievement. However, most meta-analyses found that training methods lead only to weak, non-lasting effects on cognitive control^73–75^. One meta-analysis did find evidence of near-transfer following EF training in children (in particular working memory, inhibitory control and cognitive flexibility), but found no evidence of far-transfer^20^. According to this study, training on one component leads to improved abilities in that same component but not in other EF components. Regarding adults, however, one meta-analysis suggests that EF training in general and working memory training specifically may both lead to significant near- and far-transfer effects^76^. On a neural level, a meta-analysis showed that cognitive training resulted in decreased brain activity in brain regions associated with EF^77^. According to the authors, this indicates that “training interventions reduce demands on externally focused attention” (p. 193)^77^.

With regards to meta-knowledge, several studies have reported increased task-related metacognitive abilities after training. For example, researchers found that subjects who received feedback on their metacognitive judgements regarding a perceptual decision-making task displayed better metacognitive accuracy, not only in the trained task but also in an untrained memory task^78^. Related, Baird and colleagues^79^ found that a two-week mindfulness meditation training lead to enhanced meta-knowledge in the memory domain, but not the perceptual domain. The authors link these results to evidence of increased grey matter density in the aPFC in meditation practitioners.

### Summary

Research on metacognition in cognitive science has mainly been studied through the lens of metacognitive judgements and EF (specifically performance monitoring and cognitive control). Meta-knowledge is commonly activated in subjects by asking them to rate their confidence in having successfully performed a task. A distinction is made between metacognitive sensitivity, metacognitive bias and metacognitive efficacy. Monitoring and regulating processes in EF are mainly operationalised with behavioural tasks such as Flanker tasks, Stroop tasks, Motion Discrimination tasks and Demand Selection tasks. In addition, metacognitive judgements can be viewed as offline processes in that they require the subject to reflect on her cognition and develop meta-representations. In contrast, EF can be considered as mostly online metacognitive processes because monitoring and regulation mostly happen rapidly without the need for reflective thinking.

Although there is some evidence for domain specificity, other studies have suggested that there is a single network of regions involved in all meta-cognitive tasks, but differentially activated in different task contexts. Comparing research on meta-knowledge and meta-control also suggest that some regions play a crucial role in both knowledge and regulation (Fig. 5). We have also identified a specific set of regions that are involved in either offline or online meta-knowledge. The evidence in favour of metacognitive training, while mixed, is interesting. In particular, research on offline meta-knowledge training involving self-reflection and metacognitive accuracy has shown some promising results. The regions that show structural changes after training, were those that we earlier identified as being part of the metacognition network. EF training does seem to show far-transfer effects at least in adults, but the relevance for everyday life activity is still unclear.

One major limitation of current research in metacognition is ecological validity. It is unclear to what extent the operationalisations reviewed above reflect real-life metacognition. For instance, are people who can accurately judge their performance on a behavioural task also able to accurately assess how they performed during an exam? Are people with high levels of error regulation and inhibitory control able to learn more efficiently? Note that criticism on the ecological validity of neurocognitive operationalisations extends beyond metacognition research^16^. A solution for improving validity may be to compare operationalisations of metacognition in cognitive neuroscience with the ones in educational sciences, which have shown clear links with learning in formal education. This also applies to metacognitive training.

## Metacognition in educational sciences

### Operational definitions

The most popular protocols used to measure metacognition in educational sciences are self-report questionnaires or interviews, learning journals and thinking-aloud protocols^31,80^. During interviews, subjects are asked to answer questions regarding hypothetical situations^81^. In learning journals, students write about their learning experience and their thoughts on learning^82,83^. In thinking-aloud protocols, subjects are asked to verbalise their thoughts while performing a problem-solving task^80^. Each of these instruments can be used to study meta-knowledge and meta-control. For instance, one of the most widely used questionnaires, the Metacognitive Awareness Inventory (MAI)^42^, operationalises “Flavellian” metacognition and has dedicated scales for meta-knowledge and meta-control (also popular are the MSLQ^84^ and LASSI^85^ which operate under SRL). The meta-knowledge scale of the MAI operationalises knowledge of strategies (e.g., “*I am aware of what strategies I use when I study*”) and self-awareness (e.g., “*I am a good judge of how well I understand something*”); the meta-control scale operationalises planning (e.g., “*I set a goal before I begin a task*”) and use of learning strategies (e.g., “*I summarize what I’ve learned after I finish*”). Learning journals, self-report questionnaires and interviews involve offline metacognition. Thinking aloud, though not engaging the same degree self-reflection, also involves offline metacognition in the sense that *online* processes are verbalised, which necessitate *offline* processing (see Table 1 for an overview and Supplementary Table 2 for more details).

More recently, methodologies borrowed from cognitive neuroscience have been introduced to study EF in educational settings^22,86^. In particular, researchers used classic cognitive control tasks such as the Stroop task (for a meta-analysis^86^). Most of the studied components are related to meta-control and not meta-knowledge. For instance, the BRIEF^87^ is a questionnaire completed by parents and teachers which assesses different subdomains of EF: (1) inhibition, shifting, and emotional control which can be viewed as online metacognitive control, and (2) planning, organisation of materials, and monitoring, which can be viewed as offline meta-control^87^.

Assessment of metacognition is usually compared against metrics of academic performance such as grades or scores on designated tasks. A recent meta-analysis reported a weak correlation of self-report questionnaires and interviews with academic performance whereas think-aloud protocols correlated highly^88^. Offline meta-knowledge processes operationalised by learning journals were found to be positively associated with academic achievement when related to reflection on learning activities but negatively associated when related to reflection on learning materials, indicating that the type of reflection is important^89^. EF have been associated with abilities in mathematics (mainly) and reading comprehension^86^. However, the literature points towards contrary directions as to what specific EF component is involved in academic achievement. This may be due to the different groups that were studied, to different operationalisations or to different theoretical underpinnings for EF^86^. For instance, online and offline metacognitive processes, which are not systematically distinguished in the literature, may play different roles in academic achievement. Moreover, the bulk of research focussed on young children with few studies on adolescents^86^ and EF may play a role at varying extents at different stages of life.

### Training metacognition

A critical question in educational sciences is that of the nature of the relationship between metacognition and academic achievement to understand whether learning at school can be enhanced by training metacognitive abilities. Does higher metacognition lead to higher academic achievement? Do these features evolve in parallel? Developmental research provides valuable insights into the formation of metacognitive abilities that can inform training designs in terms of what aspect of metacognition should be supported and the age at which interventions may yield the best results. First, meta-knowledge seems to emerge around the age of 5, meta-control around 8, and both develop over the years^90^, with evidence for the development of meta-knowledge into adolescence^91^. Furthermore, current theories propose that meta-knowledge abilities are initially highly domain-dependent and gradually become more domain-independent as knowledge and experience are acquired and linked between domains^32^. Meta-control is believed to evolve in a similar fashion^90,92^.

Common methods used to train offline metacognition are direct instruction of metacognition, metacognitive prompts and learning journals. In addition, research has been done on the use of (self-directed) feedback as a means to induce self-reflection in students, mainly in computer-supported settings^93^. Interestingly, learning journals appear to be used for both assessing and fostering metacognition. Metacognitive instruction consists of teaching learners’ strategies to “activate” their metacognition. Metacognitive prompts most often consist of text pieces that are sent at specific times and that trigger reflection (offline meta-knowledge) on learning behaviour in the form of a question, hint or reminder.

Meta-analyses have investigated the effects of direct metacognitive instruction on students’ use of learning strategies and academic outcomes^18,94,95^. Their findings show that metacognitive instruction can have a positive effect on learning abilities and achievement within a population ranging from primary schoolers to university students. In particular, interventions lead to the highest effect sizes when they both (i) instructed a combination of metacognitive strategies with an emphasis on planning strategies (offline meta-control) and (ii) “provided students with knowledge about strategies” (offline meta-knowledge) and “illustrated the benefits of applying the trained strategies, or even stimulated metacognitive reasoning” (p.114)^18^. The longer the duration of the intervention, the more effective they were. The strongest effects on academic performance were observed in the context of mathematics, followed by reading and writing.

While metacognitive prompts and learning journals make up the larger part of the literature on metacognitive training^96^, meta-analyses that specifically investigate their effectiveness have yet to be performed. Nonetheless, evidence suggests that such interventions can be successful. Researchers found that metacognitive prompts fostered the use of metacognitive strategies (offline meta-control) and that the combination of cognitive and metacognitive prompts improved learning outcomes^97^. Another experiment showed that students who received metacognitive prompts performed more metacognitive activities inside the learning environment and displayed better transfer performance immediately after the intervention^98^. A similar study using self-directed prompts showed enhanced transfer performance that was still observable 3 weeks after the intervention^99^.

Several studies suggest that learning journals can positively enhance metacognition. Subjects who kept a learning journal displayed stronger high meta-control and meta-knowledge on learning tasks and tended to reach higher academic outcomes^100–102^. However, how the learning journal is used seems to be critical; good instructions are crucial^97,103^, and subjects who simply summarise their learning activity benefit less from the intervention than subjects who reflect about their knowledge, learning and learning goals^104^. An overview of studies using learning journals and metacognitive prompts to train metacognition can be found in Supplementary Table 3.

In recent years, educational neuroscience researchers have tried to determine whether training and improvements in EF can lead to learning facilitation and higher academic achievement. Training may consist of having students continually perform behavioural tasks either in the lab, at home, or at school. Current evidence in favour of training EF is mixed, with only anecdotal evidence for positive effects^105^. A meta-analysis did not show evidence for a causal relationship between EF and academic achievement^19^, but suggested that the relationship is bidirectional, meaning that the two are “mutually supportive”^106^.

A recent review article has identified several gaps and shortcoming in the literature on metacognitive training^96^. Overall, research in metacognitive training has been mainly invested in developing learners’ meta-control rather than meta-knowledge. Furthermore, most of the interventions were done in the context of science learning. Critically, there appears to be a lack of studies that employed randomised control designs, such that the effects of metacognitive training intervention are often difficult to evaluate. In addition, research overwhelmingly investigated metacognitive prompts and learning journals in adults^96^, while interventions on EF mainly focused on young children^22^. Lastly, meta-analyses evaluating the effectiveness of metacognitive training have so far focused on metacognitive instruction on children. There is thus a clear disbalance between the meta-analyses performed and the scope of the literature available.

An important caveat of educational sciences research is that metacognition is not typically framed in terms of online and offline metacognition. Therefore, it can be unclear whether protocols operationalise online or offline processes and whether interventions tend to benefit more online or offline metacognition. There is also confusion in terms of what processes qualify as EF and definitions of it vary substantially^86^. For instance, Clements and colleagues mention work on SRL to illustrate research in EF in relation to academic achievement but the two spawn from different lines of research, one rooted in metacognition and socio-cognitive theory^31^ and the other in the cognitive (neuro)science of decision-making. In addition, the MSLQ, as discussed above, assesses *offline* metacognition along with other components relevant to SRL, whereas EF can be mainly understood as *online* metacognition (see Table 1), which on the neural level may rely on different circuitry.

### Summary

Investigating offline metacognition tends to be carried out in school settings whereas evaluating EF (e.g., Stroop task, and BRIEF) is performed in the lab. Common to all protocols for offline metacognition is that they consist of a form of self-report from the learner, either during the learning activity (thinking-aloud protocols) or after the learning activity (questionnaires, interviews and learning journals). Questionnaires are popular protocols due to how easy they are to administer but have been criticised to provide biased evaluations of metacognitive abilities. In contrast, learning journals evaluate the degree to which learners engage in reflective thinking and may therefore be less prone to bias. Lastly, it is unclear to what extent thinking-aloud protocols are sensitive to online metacognitive processes, such as on-the-fly error correction and effort regulation. The strength of the relationship between metacognitive abilities and academic achievement varies depending on how metacognition is operationalised. Self-report questionnaires and interviews are weakly related to achievement whereas thinking-aloud protocols and EF are strongly related to it.

Based on the well-documented relationship between metacognition and academic achievement, educational scientists hypothesised that fostering metacognition may improve learning and academic achievement, and thus performed metacognitive training interventions. The most prevalent training protocols are direct metacognitive instruction, learning journals, and metacognitive prompts, which aim to induce and foster offline metacognitive processes such as self-reflection, planning and selecting learning strategies. In addition, researchers have investigated whether training EF, either through tasks or embedded in the curriculum, results in higher academic proficiency and achievement. While a large body of evidence suggests that metacognitive instruction, learning journals and metacognitive prompts can successfully improve academic achievement, interventions designed around EF training show mixed results. Future research investigating EF training in different age categories may clarify this situation. These various degrees of success of interventions may indicate that offline metacognition is more easily trainable than online metacognition and plays a more important role in educational settings. Investigating the effects of different methods, offline and online, on the neural level, may provide researchers with insights into the trainability of different metacognitive processes.

## Discussion

In this article, we reviewed the literature on metacognition in educational sciences and cognitive neuroscience with the aim to investigate gaps in current research and propose ways to address them through the exchange of insights between the two disciplines and interdisciplinary approaches. The main aspects analysed were operational definitions of metacognition and metacognitive training, through the lens of metacognitive knowledge and metacognitive control. Our review also highlighted an additional construct in the form of the distinction between online metacognition (on the fly and largely automatic) and offline metacognition (slower, reflective and requiring meta-representations). In cognitive neuroscience, research has focused on metacognitive judgements (mainly offline) and EF (mainly online). Metacognition is operationalised with tasks carried out in the lab and are mapped onto brain functions. In contrast, research in educational sciences typically measures metacognition in the context of learning activities, mostly in schools and universities. More recently, EF has been studied in educational settings to investigate its role in academic achievement and whether training it may benefit learning. Evidence on the latter is however mixed. Regarding metacognitive training in general, evidence from both disciplines suggests that interventions fostering learners’ self-reflection and knowledge of their learning behaviour (i.e., offline meta-knowledge) may best benefit them and increase academic achievement.

We focused on four aspects of research that could benefit from an interdisciplinary approach between the two areas: (i) validity and reliability of research protocols, (ii) under-researched dimensions of metacognition, (iii) metacognitive training, and (iv) domain-specificity vs. domain generality of metacognitive abilities. To tackle these issue, we propose four avenues for integrated research: (i) investigate the degree to which different protocols relate to similar or different metacognitive constructs, (ii) implement designs and perform experiments to identify neural substrates necessary for offline meta-control by for example borrowing protocols used in educational sciences, (iii) study the effects of (offline) meta-knowledge training on the brain, and (iv) perform developmental research in the metacognitive brain and compare it with the existing developmental literature in educational sciences regarding the domain-generality of metacognitive processes and metacognitive abilities.

First, neurocognitive research on metacognitive judgements has developed robust operationalisations of offline meta-knowledge. However, these operationalisations often consist of specific tasks (e.g., 2-AFC) carried out in the lab. These tasks are often very narrow and do not resemble the challenges and complexities of behaviours associated with learning in schools and universities. Thus, one may question to what extent they reflect real-life metacognition, and to what extent protocols developed in educational sciences and cognitive neuroscience actually operationalise the same components of metacognition. We propose that comparing different protocols from both disciplines that are, a priori, operationalising the same types of metacognitive processes can help evaluate the ecological validity of protocols used in cognitive neuroscience, and allow for more holistic assessments of metacognition, provided that it is clear which protocol assesses which construct. Degrees of correlation between different protocols, within and between disciplines, may allow researchers to assess to what extent they reflect the same metacognitive constructs and also identify what protocols are most appropriate to study a specific construct. For example, a relation between meta-*d*′ metacognitive sensitivity in a 2-AFC task and the meta-knowledge subscale of the MAI, would provide external validity to the former. Moreover, educational scientists would be provided with bias-free tools to assess metacognition. These tools may enable researchers to further investigate to what extent metacognitive bias, sensitivity and efficiency each play a role in education settings. In contrast, a low correlation may highlight a difference in domain between the two measures of metacognition. For instance, metacognitive judgements in brain research are made in isolated behaviour, and meta-d’ can thus be viewed to reflect “local” metacognitive sensitivity. It is also unclear to what extent processes involved in these decision-making tasks cover those taking place in a learning environment. When answering self-reported questionnaires, however, subjects make metacognitive judgements on a large set of (learning) activities, and the measures may thus resemble more “global” or domain-general metacognitive sensitivity. In addition, learners in educational settings tend to receive feedback — immediate or delayed — on their learning activities and performance, which is generally not the case for cognitive neuroscience protocols. Therefore, investigating metacognitive judgements in the presence of performance or social feedback may allow researchers to better understand the metacognitive processes at play in educational settings. Devising a global measure of metacognition in the lab by aggregating subjects’ metacognitive abilities in different domains or investigating to what extent local metacognition may affect global metacognition could improve ecological validity significantly. By investigating the neural correlates of educational measures of metacognition, researchers may be able to better understand to what extent the constructs studied in the two disciplines are related. It is indeed possible that, though weakly correlated, the meta-knowledge scale of the MAI and meta-d’ share a common neural basis.

Second, our review highlights gaps in the literature of both disciplines regarding the research of certain types of metacognitive processes. There is a lack of research in offline meta-control (or strategic regulation of cognition) in neuroscience, whereas this construct is widely studied in educational sciences. More specifically, while there exists research on EF related to planning (e.g.^107^), common experimental designs make it hard to disentangle online from offline metacognitive processes. A few studies have implemented subject reports (e.g., awareness of error or desire for reminders) to pin-point the neural substrates specifically involved in offline meta-control and the current evidence points at a role of the lPFC. More research implementing similar designs may clarify this construct. Alternatively, researchers may exploit educational sciences protocols, such as self-report questionnaires, learning journals, metacognitive prompts and feedback to investigate offline meta-control processes in the brain and their relation to academic proficiency and achievement.

Third, there is only one study known to us on the training of meta-knowledge in the lab^78^. In contrast, meta-knowledge training in educational sciences have been widely studied, in particular with metacognitive prompts and learning journals, although a systematic review would be needed to identify the benefits for learning. Relative to cognitive neuroscience, studies suggest that offline meta-knowledge trained in and outside the lab (i.e., metacognitive judgements and meditation, respectively) transfer to meta-knowledge in other lab tasks. The case of meditation is particularly interesting since meditation has been demonstrated to beneficiate varied aspects of everyday life^108^. Given its importance for efficient regulation of cognition, training (offline) meta-knowledge may present the largest benefits to academic achievement. Hence, it is important to investigate development in the brain relative to meta-knowledge training. Evidence on metacognitive training in educational sciences tends to suggest that offline metacognition is more “plastic” and may therefore benefit learning more than online metacognition. Furthermore, it is important to have a good understanding of the developmental trajectory of metacognitive abilities — not only on a behavioural level but also on a neural level — to identify critical periods for successful training. Doing so would also allow researchers to investigate the potential differences in terms of plasticity that we mention above. Currently, the developmental trajectory of metacognition is under-studied in cognitive neuroscience with only one study that found an overlap between the neural correlates of metacognition in adults and children^109^. On a side note, future research could explore the potential role of genetic factors in metacognitive abilities to better understand to what extent and under what constraints they can be trained.

Fourth, domain-specific and domain-general aspects of metacognitive processes should be further investigated. Educational scientists have studied the development of metacognition in learners and have concluded that metacognitive abilities are domain-specific at the beginning (meaning that their quality depends on the type of learning activity, like mathematics vs. writing) and progressively evolve towards domain-general abilities as knowledge and expertise increase. Similarly, neurocognitive evidence points towards a common network for (offline) metacognitive knowledge which engages the different regions at varying degrees depending on the domain of the activity (i.e., perception, memory, etc.). Investigating this network from a developmental perspective and comparing findings with the existing behavioural literature may improve our understanding of the metacognitive brain and link the two bodies of evidence. It may also enable researchers to identify stages of life more suitable for certain types of metacognitive intervention.

## Supplementary information

Supplementary materials
