# Supplementary material for: Metacognition: ideas and insights from neuro- and educational sciences
Source: NPJ Sci Learn. 2021 Jun 8;6:13. doi: 10.1038/s41539-021-00089-5 (PMC8187395; doi:10.1038/s41539-021-00089-5)
Supplement: Supplementary file 1 — Supplementary materials [file 41539_2021_89_MOESM1_ESM.pdf]

Supplementary Table 1: Types of metacognitive processes studied in each article along with protocol and measuring instrument used, and the associated brain regions.

online vs offline	meta knowledge vs control	Process	Protocols	Brain measure	Brain regions	Article	Link
Offline	knowledge	Metacognitive Judgments	2-AFC + Confidence ratings	MRI	aPFC	Fleming et al. (2010)	https://doi.org/10.1126/science.1191883
Offline	knowledge	Metacognitive Judgments	2-AFC + Confidence ratings	MRI	aPFC, precuneus	McCurdy et al. (2013)	https://doi.org/10.1523/JNEUROSCI.1890-12.2013
Offline	knowledge	Metacognitive Judgments	2-AFC + Confidence ratings	fMRI	right rlPFC	Fleming et al. (2012)	https://doi.org/10.1523/JNEUROSCI.6489-11.2012
Offline	knowledge	Metacognitive Judgments	2-AFC + Confidence ratings	fMRI	right rlPFC, dACC	Morales et al. (2018)	https://doi.org/10.1523/JNEUROSCI.2360-17.2018
Offline	knowledge	Metacognitive Judgments	2-AFC + Confidence ratings	brain lesions	aPFC	Fleming et al. (2014)	https://doi.org/10.1093/brain/awu221
Offline	knowledge	Metacognitive Judgments	2-AFC + Confidence ratings	rs-fcMRI	connectivity lateral aPFC-right dACC and medial aPFC-precuneus	Baird et al. (2013)	https://doi.org/10.1523/JNEUROSCI.0786-13.2013
Offline	control	Error correction	Motion Discrimination task + additional evidence + confidence ratings	fMRI	lateral aPFC	Fleming et al. (2018)	https://doi.org/10.1038/s41593-018-0104-6
Offline	control	Effort regulation	Demand selection task + self-report	fMRI	right lPFC	McGuire & Botvinic (2010)	https://doi.org/10.1073/pnas.0910662107
Online	knowledge	Error/conflict detection/monitoring	Bimanual choice reaction task	EEG		Falkenstein et al. (1991)	https://doi.org/10.1016/0013-4694(91)90062-9
Online	knowledge	Error/conflict detection/monitoring	Flanker task	EEG + fMRI	rostral cingulate zone	Debener et al. (2005)	https://doi.org/10.1523/JNEUROSCI.3286-05.2005
Online	knowledge	Error/conflict detection/monitoring	Flanker task	EEG		Nieuwenhuis et al. (2001)	https://doi.org/10.1111/1469-8986.3850752
Online	knowledge	Error/conflict detection/monitoring	Flanker task	EEG + fMRI	ACC	Overbeek et al. (2005)	https://doi.org/10.1027/0269-8803.19.4.319
Online	knowledge	Error/conflict detection/monitoring	Stroop task	PET	ACC	Bench et al. (1993)	http://hdl.handle.net/21.11116/0000-0001-A38B-A
Online	knowledge	Error/conflict detection/monitoring	Simon task	fMRI	ACC	Witthof et al. (2008)	https://doi.org/10.1016/j.brainres.2007.11.067
Online	knowledge	Effort monitoring	Demand selection task + self-report	fMRI	dfMC	McGuire & Botvinic (2010)	https://doi.org/10.1073/pnas.0910662107
Online	control	Conflict resolution	Design fluency + verbal fluency	brain lesions	left PFC	Baldo et al. (2001)	https://psycnet.apa.org/doi/10.1017/S1355617701755063
Online	control	Effort regulation	Delayed-response task	fMRI	dlPFC	D'Esposito et al. (2000)	https://doi.org/10.1007/978-3-642-59794-7_2

Supplementary Table 2: Overview of studies illustrating how metacognition is operationalised using certain protocols.

meta-knowlege vs control	Protocols	Article	Link
knowledge	Learning journals	Dianovsky and Wink (2012)	https://doi.org/10.1002/sce.21010
knowledge + control	Learning journals	Nückles et al. (2020)	https://doi.org/10.1007/s10648-020-09541-1
knowledge + control	Learning journals	Schmitz and Wiese (2006)	https://doi.org/10.1016/j.cedpsych.2005.02.002
knowledge + control	Learning journals	Berthold et al. (2007)	https://doi.org/10.1016/j.learninstruc.2007.09.007
knowledge + control	Learning journals	Otto and Kistner (2017)	https://doi.org/10.1016/j.lindif.2017.03.005
knowledge + control	Learning journals, self-report questionnaire, thinking-aloud	Jacobse and Harskamp (2012)	https://doi.org/10.1007/s11409-012-9088-x
knowledge + control	Learning journals	Larkin (2009)	https://doi.org/10.1016/j.tsc.2009.09.003
knowledge + control	Learning journals	Ferreira et al (2015)	https://doi.org/10.1007/s11409-014-9121-3
knowledge + control	Learning journals	Wallin and Adawi (2018)	https://doi.org/10.1080/03043797.2017.1290585
knowledge	Learning journals	Ramandhanti et al. (2020)	https://www.learntechlib.org/p/217098/
knowledge + control	Learning journals	Andreassen et al. (2017)	https://doi.org/10.1007/s11145-017-9758-9
knowledge + control	Learning journals	Lew and Schmidt (2011)	https://doi.org/10.1080/07294360.2010.512627
knowledge	Learning journals	Klug et al. (2018)	http://www.sciencedirect.com/science/article/pii/S0742051X17302937
knowledge + control	Self-report questionnaire: MAI	Schraw and Dennison (1994)	https://doi.org/10.1006/ceps.1994.1033
knowledge + control	Self-report questionnaire: MAI	Harrison and Vallin (2018)	https://doi.org/10.1007/s11409-017-9176-z
knowledge + control	Interviews	Zimmerman and Martinez-Pons (1986)	https://doi.org/10.3102%2F00028312023004614
knowledge	Interviews	Semerari et al. (2012)	https://doi.org/10.1016/j.psychres.2012.07.015
knowledge	Interviews	Craig and Yore (1995)	https://doi.org/10.1080/0270271950160203
knowledge	Interviews	Marulis et al. (2016)	https://doi.org/10.1007/s11409-016-9157-7
knowledge	Interviews	Myers and Paris (1978)	https://psycnet.apa.org/doi/10.1037/0022-0663.70.5.680
control	Thinking-aloud protocols	Azevedo et al (2008)	https://doi.org/10.1007/s11423-007-9067-0
control	Thinking-aloud protocols	van der Stel and Veenman (2010)	https://doi.org/10.1016/j.lindif.2009.11.005
control	Thinking-aloud protocols	Veenman and Beishuizen (2004)	https://doi.org/10.1016/j.learninstruc.2004.09.004
control	Self-report questionnaire: MSLQ	Pintrich et al. (1993)	https://doi.org/10.1177/0013164493053003024
control	Self-report questionnaire: LASSI	Prevatt et al. (2006)	https://doi.org/10.1177%2F0013164405282454
control	Stroop Task	review: Baggetta and Alexander (2016)	https://doi.org/10.1111/mbe.12100
control	D-KEFS	review: Baggetta and Alexander (2016)	https://doi.org/10.1111/mbe.12100
control	BRIEF	review: Baggetta and Alexander (2016)	https://doi.org/10.1111/mbe.12100

Supplementary Table 3: Overview of metacognitive training studies along with the type of intervention performed and outcome measures.

Type of intervention	Outcome measure	Article	Link
Learning journal	MSLQ	Arsal (2010)	https://eric.ed.gov/?id=EJ884413
Metacognitive prompts	learning outcomes + self-report questionnaire, video-analysis	Bannert and Mengelkamp (2013)	https://doi.org/10.1007/978-1-4419-5546-3_12
Metacognitive prompt	learning outcome, learning journal	Berthold et al. (2007)	htps://doi.org/10.1016/j.learninstruc.2007.09.007
Learning journal	grades	Connor-Greene (2000)	https://doi.org/10.1207/S15328023TOP2701_10
Learning journal	grades + self-report quetionnaire + learning journals	Dianovski and Wink (2012)	https://doi.org/10.1002/sce.21010
Learning journal	self-report questionnaire, learning journal	Ewijk et al. (2015)	https://doi.org/10.1891/1945-8959.14.1.77
Learning journal	grades, self-report questionnaire, test	Fabriz et al. (2014)	https://doi.org/10.1007/s10212-013-0196-z
Learning journal	self-report questionnaire, interview, learning strategies task, grades	McCrindle and Christensen (1995)	https://doi.org/10.1016/0959-4752(95)00010-Z
Learning journal + prompts	learning journals, learning outcomes	Nückles et al. (2020)	https://doi.org/10.1007/s10648-020-09541-1
Learning journal	Learning journal	Otto and Kistner (2017)	https://doi.org/10.1016/j.lindif.2017.03.005
Learning journal	learning outcomes	Park (2003)	https://doi.org/10.1080/03098260305675
Learning journal	learning journals, Self-report questionnaires	Schmitz and Wiese (2006)	https://doi.org/10.1016/j.cedpsych.2005.02.002
Learning journal	reading comprehension test, interviews	Wong et al. (2002)	https://doi.org/10.1080/00220670209596588
Learning journal + prompts	knowledge test, learning journal,	Petko et al. (2014)	http://earthlab.uoi.gr/theste/index.php/theste/article/view/146
Learning journal	learning journal, self-report questionaire, math test	Schmitz and Perels (2011)	http://dx.doi.org/10.1007%2Fs11409-011-9076-6
Learning journal	MSLQ, Declarative knowledge test	Boradbent et al. (2020)	https://doi.org/10.1007/s11423-020-09781-6
Learning journal	grades, MSLQ	Cazan (2012)	https://doi.org/10.1016/j.sbspro.2012.01.154
Learning journal	MAI, MSLQ	Cho et al. (2017)	https://doi.org/10.14742/ajet.2996
Learning journal	self-report questionnaire	Dörrenbächer and Perels (2016)	https://doi.org/10.1016/j.ijer.2016.05.010
Learning journal + prompts	MSLQ, learning journal	Fung et al. (2019)	https://eric.ed.gov/?id=EJ1239150
Learning journal	MSLQ	Guvenc (2010)	https://eric.ed.gov/?id=EJ919859
Metacognitive prompt	academic performance	Davis et al. (2016)	https://doi.org/10.1007/978-3-319-45153-4_5
Metacognitive prompt	learning outcomes	Wong et al. (2019)	https://doi.org/10.1016/j.compedu.2019.103595
Metacognitive prompt	MSLQ	Van den Boom et al. (2007)	https://doi.org/10.1016/j.learninstruc.2007.09.003
Metacognitive prompt	MSLQ	Van den Boom et al. (2004)	https://doi.org/10.1016/j.chb.2003.10.001
Metacognitive prompt	logfliles	Thillmann et al. (2009)	https://doi.org/10.1024/1010-0652.23.2.105
Metacognitive prompt	logfliles	Taub et al. (2014)	https://doi.org/10.1016/j.chb.2014.07.018
Metacognitive prompt	academic achievement	Mevarech and Kramarski (1997)	https://doi.org/10.3102/00028312034002365
Metacognitive prompt	learning outcomes	Long and Aleven (2013)	https://doi.org/10.1007/978-3-642-39112-5_23
Metacognitive prompt	MAI, Domain-specific knowledge test	Lehman et al. (2014)	https://doi.org/10.1016/j.chb.2013.07.051
Metacognitive prompts	academic performance	Kramarski et al. (2010)	http://dx.doi.org/10.1007%2Fs11858-009-0202-8
Metacognitive prompt	aptitude test, MSLQ	Kramarski and Michalsky (2010)	https://doi.org/10.1016/j.learninstruc.2009.05.003
Metacognitive prompt	academic achievement, note taking	Kauffman et al. (2011)	https://doi.org/10.1016/j.cedpsych.2011.04.001
Metacognitive prompt	academic achievement	Kauffman (2004)	https://doi.org/10.2190/AX2D-Y9VM-V7PX-0TAD
Metacognitive prompt	academic performance, self-report questionnaire	Jones and Castellano (2018)	https://doi.org/10.1007/s12369-017-0458-z
Metacognitive prompt	academic performance	Jones et al. (2018)	https://doi.org/10.1007/s12369-017-0430-y
Learning journal	self-report questionnaire	Jado (2015)	https://eric.ed.gov/?id=EJ1083603
Metacognitive prompt	self-report questionnaire, MAI, academic performance	Ifenthaler (2012)	https://eric.ed.gov/?id=EJ979448
Metacognitive prompt	Logfiles, academic achievement	Duffy and Azevedo (2015)	https://doi.org/10.1016/j.chb.2015.05.041
Metacognitive prompt	learning efficiency, log file	Azevedo et al. (2012)	https://doi.org/10.1007/978-3-642-30950-2_27
Metacognitive prompt	thinking-aloud,	Azevedo et al. (2008)	https://doi.org/10.1007/s11423-007-9067-0
